# Propagation of Plasma L-Phenylalanine Concentration Fluctuations to the Neurovascular Unit in Phenylketonuria: An *in silico* Study

**DOI:** 10.3389/fphys.2019.00360

**Published:** 2019-04-02

**Authors:** Mehdi Taslimifar, Stefano Buoso, Francois Verrey, Vartan Kurtcuoglu

**Affiliations:** ^1^The Interface Group, Institute of Physiology, University of Zurich, Zurich, Switzerland; ^2^Epithelial Transport Group, Institute of Physiology, University of Zurich, Zurich, Switzerland; ^3^Institute for Diagnostic and Interventional Radiology, Zurich University Hospital, Zurich, Switzerland; ^4^Zurich Center for Integrative Human Physiology, University of Zurich, Zurich, Switzerland; ^5^National Center of Competence in Research, Kidney Control of Homeostasis, Zurich, Switzerland; ^6^Neuroscience Center Zurich, University of Zurich, Zurich, Switzerland

**Keywords:** phenylketonuria, L-phenylalanine fluctuation, neurovascular unit, amino acid transporter, large neutral amino acid

## Abstract

Phenylketonuria (PKU) is an inherited metabolic disease characterized by abnormally high concentrations of the essential amino acid L-phenylalanine (Phe) in blood plasma caused by reduced activity of phenylalanine hydroxylase (PAH). While numerous studies have shown association between high plasma Phe concentration and intellectual impairment, it is not clear whether increased Phe fluctuations also observed in PKU affect the brain as well. To investigate this, time-resolved *in vivo* data on Phe and competing large neutral amino acid (LNAA) concentrations in neurons are needed, but cannot be acquired readily with current methods. We have used *in silico* modeling as an alternative approach to characterize the interactive dynamics of Phe and competing LNAAs (CL) in the neurovascular unit (NVU). Our results suggest that plasma Phe fluctuations can propagate into the NVU cells and change there the concentration of LNAAs, with the highest magnitude of this effect observed at low frequency and high amplitude-to-mean ratio of the plasma Phe concentration fluctuations. Our model further elucidates the effect of therapeutic LNAA supplementation in PKU, showing how abnormal concentrations of Phe and CL in the NVU move thereby toward normal physiologic levels.

## Introduction

Phenylketonuria (PKU) is the most common disorder of amino acid (AA) metabolism, resulting from severely reduced activity of the liver enzyme phenylalanine hydroxylase (PAH), which leads to abnormal accumulation of the essential amino acid L-phenylalanine (Phe) in the blood plasma (Güttler et al., 1969; Madden, 2004; Regier and Greene, 2017). Phe is transported across the blood brain barrier (BBB) into the neurovascular unit (NVU), where abnormal increase in its concentration leads to imbalance of large neutral amino acid (LNAA) levels in brain interstitial fluid (ISF), astrocytes and neurons, as a consequence of the competition for NVU-LNAA transporters (van Spronsen et al., 2010). Since NVU-LNAAs are required for the synthesis of essential neurotransmitters such as dopamine, serotonin, and norepinephrine, their perturbations can lead to intellectual disabilities, growth abnormalities and severe behavioral problems in PKU patients (Güttler et al., 1969; Cleary et al., 2013).

The dysfunction of the PAH enzyme is also the cause of higher than normal fluctuations in Phe plasma concentration, where patterns greatly vary among patients in relation to age, diet, genotype and level of PAH defect (Burgard et al., 1996; Cleary et al., 2013). Nevertheless, only time-averaged Phe concentrations are used to classify PKU severity (benign, mild or classic) and design the treatment protocol, which consists mainly of a low Phe diet to reduce mean blood Phe concentration (Cleary et al., 2013; Belanger et al., 2018). However, the effectiveness of such therapies depends on the concentrations of LNAAs in the NVU, which can influence brain function and behavior, rather than directly the blood Phe level (Cleary et al., 2013). Some studies have specifically investigated the relation between brain function (assessed through intelligence quotient (IQ) tests) and mean plasma Phe concentration and its fluctuations. The results were not conclusive: while in some studies IQ was found to be more often associated with the level of Phe fluctuations in the plasma rather than with its mean value (Hood et al., 2014; Romani et al., 2017), the opposite was reported in others (Viau et al., 2011; Hood et al., 2015), while no differentiation was possible in yet another one (Burgard et al., 1996). The reasons for this inconsistency are not clear, but could be, in part, a reflection of how fluctuations in plasma Phe concentration translate in individual patients to effects on AA homeostasis in the brain (Cleary et al., 2013).

**FIGURE 1:**
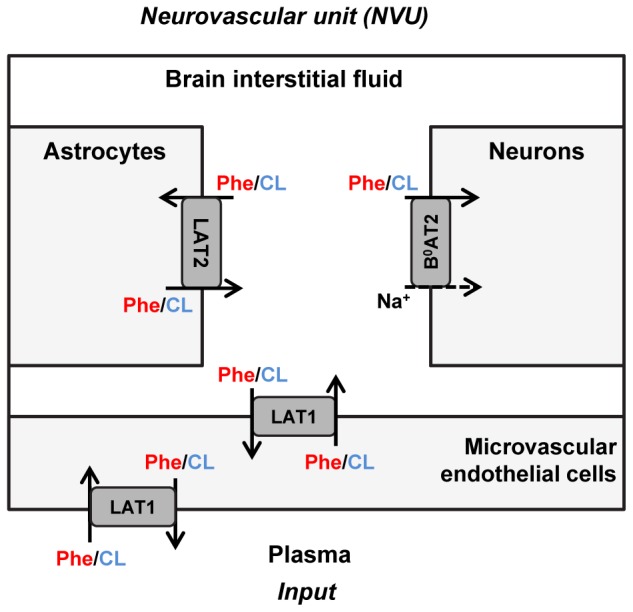
Schematic representation of the neurovascular unit and the therein expressed dominant Phe and CL transporters. The dominant Phe and competing large neutral amino acid (CL) transporters involved in the pathophysiology of PKU disorder include the Na^+^-independent antiporter LAT1 (SLC7A5) in microvascular brain endothelial cells (MBECs), the Na^+^-independent antiporter LAT2 (SLC7A8) in astrocytes and the Na^+^-dependent symporter B^0^AT2 (SLC6A15) in neurons. The arrows specify Phe and CL transmembrane pathways. The plasma concentration profiles of Phe and CL are taken as the input to the neurovascular unit (NVU), based on which concentrations in the NVU compartments are calculated.

The propagation of Phe fluctuations from plasma into the brain is critically influenced by the competition between this AA and its competing LNAAs (CL, i.e., L-leucine, L-isoleucine, L-tyrosine, L-tryptophan, L-valine, L-histidine, and L-methionine) for transporters at NVU cell membranes (Taslimifar et al., 2018). It has been shown that the Na^+^-independent antiporter SLC7A5 (LAT1) in microvascular brain endothelial cells (MBEC) (Smith et al., 1987; Killian and Chikhale, 2001; Meier et al., 2002), the Na^+^-independent antiporter SLC7A8 (LAT2) in astrocytes (Yudkoff et al., 1996a; Kim et al., 2004; Braun et al., 2011), and the Na^+^-dependent symporter SLC6A15 (B^0^AT2) in neurons (Yudkoff et al., 1996b; Bröer et al., 2006; Bak et al., 2012) are the main regulators of LNAAs homeostasis in the NVU (Figure 1). *In vivo* monitoring of LNAA levels in individual NVU compartments during fluctuations of plasma Phe concentrations could help establish a better understanding of their interrelation, but there are technological hurdles that need to be overcome to enable corresponding experiments.

To go around these hurdles, we have employed a previously developed computational model of NVU-LNAAs homeostasis (Taslimifar et al., 2018) to explore *in silico* how plasma Phe fluctuations influence LNAA concentrations in the NVU. In addition, we have quantified the variations in concentration of Phe and CL in NVU cells in relation to descriptors of plasma Phe fluctuation, namely mean, fundamental frequency and amplitude-to-mean ratio (Anastasoaie et al., 2008; Cleary et al., 2013). Finally, we have employed the model to explore the impact of therapeutic supplementation of LNAAs on the attenuation of Phe and CL concentrations in NVU cells. While this treatment strategy has been shown to modulate the perturbed concentration of LNAAs in brain tissue in a PKU mouse model (van Vliet et al., 2015, 2016) and also to positively impact executive functioning of PKU patients (Schindeler et al., 2007; van Spronsen et al., 2010), it remains unclear how supplementation of LNAAs affects the dynamics of Phe and CL concentration in MBECs, astrocyte and more importantly in neurons (van Spronsen et al., 2010; van Vliet et al., 2016).

## Materials and Methods

### Transport Model

We employed a previously developed compartmental model of NVU-LNAA homeostasis in adult rats (Taslimifar et al., 2018), to which we refer the reader for detail. Four individual NVU compartments are considered – MBECs, ISF, astrocytes (ast), and neurons (neu) – within which LNAAs are assumed to be homogeneously distributed (Figure 1). In the model, LNAA fluxes between compartments are mediated by the dominant transporters identified from literature, i.e., LAT1, LAT2, and B^0^AT2 (Table 1). Fluxes mediated by the antiporters LAT1 and LAT2 are a function of the following: maximum transport rates at the BBB luminal (lum) (V_max,LAT1,lum_) and abluminal (abl) (V_max,LAT1,abl_) membranes and astrocyte membrane (V_max,LAT2_), respectively; Michaelis-Menten binding constants in the individual NVU compartments (${\text{K}}_{\text{m,LAT}1}^{\text{P}(\text{MBEC,ISF})}$ and ${\text{K}}_{\text{m,LAT}2}^{\text{ISF}(\text{Ast})}$); and the LAT1 bi-directional kinetic constant (RK_LAT1_), which corresponds to the ratio between the absolute Michaelis-Menten binding constant for LAT1 in MBECs relative to the corresponding value in the ISF and in plasma ($\frac{{\text{K}}_{\text{m,abs,LAT1}}^{\text{MBEC}}}{{\text{K}}_{\text{m,abs,LAT1}}^{\text{P}(ISF)}}$) (Taslimifar et al., 2018). The fluxes mediated by B^0^AT2 symporter are a function of Michaelis-Menten kinetic parameters (V_max,B^0^AT2_ and ${\text{K}}_{{\text{m,B}}^{0}\text{AT}2}^{\text{ISF}(\text{Neu})}$); neuronal electrical potential-induced biases for forward and backward transport rates (ε and ε′); neuronal potential difference (Δψ), electrical bias constant (β), Faraday constant (F), sodium charge (Z), gas constant (R), and absolute temperature (T) [see (Taslimifar et al., 2018) for details]. To account for the approximate 1:1 stoichiometry observed for LAT1 and LAT2 antiporters under normal physiologic conditions (Meier et al., 2002; Verrey, 2003), at each instant we limited Phe and CL fluxes to the lowest value between the two. The values of kinetic parameters and compartment volumes (V_i_) used in the model are taken from literature, and are reported in Table 1. The same values are considered for both PKU and normal physiologic cases (Möller et al., 1997; Taslimifar et al., 2018).

**Table 1:** Model parameters.

	Value	Unit	Reference
**LAT1 (MBEC)**			
${\text{K}}_{\text{m,abs,LAT}1,\text{Phe}}^{\text{P}(\text{ISF})}$	11	μM	Smith et al., 1987
V_max,LAT1,lum,Phe_	0.075	μmol/min	Smith et al., 1987; Tilgmann et al., 1992
${\text{K}}_{\text{m,abs,LAT}1,CL}^{\text{P}(\text{ISF})}$	52.9^∗^	μM	Smith et al., 1987
V_max,LAT1,lum,CL_	0.129^∗^	μmol/min	Smith et al., 1987; Tilgmann et al., 1992
RK_LAT1_	80	–	Taslimifar et al., 2018
**LAT2 (ASTROCYTE)**			
${\text{K}}_{\text{m,abs,LAT}2,\text{Phe}}^{\text{ISF}(Ast)}$	110.2^∗^	μM	Kim et al., 2004
V_max,LAT2,Phe_	0.1128	μmol/min	Shank and Campbell, 1984; Segawa et al., 1999
${\text{K}}_{\text{m,abs,LAT}2,\text{CL}}^{\text{ISF}(Ast)}$	185.9^∗^	μM	Kim et al., 2004
V_max,LAT2,CL_	0.1494^∗^	μmol/min	Shank and Campbell, 1984; Segawa et al., 1999
**B0AT2 (NEURON)**			
${\text{K}}_{{\text{m,abs,B}}^{0}\text{AT}2,\text{Phe}}^{\text{ISF}(\text{Neu})}$	1050	μM	Bröer et al., 2006
V_max,B^0^AT2,Phe_	0.0086^∗^	μmol/min	Rao et al., 1995; Bröer et al., 2006
${\text{K}}_{{\text{m,abs,B}}^{0}\text{AT}2,\text{CL}}^{\text{ISF}(\text{Neu})}$	126.2^∗^	μM	Bröer et al., 2006
V_max,B^0^AT2,CL_	0.0186^∗^	μmol/min	Rao et al., 1995; Bröer et al., 2006
${\text{K}}_{{\text{m,B}}^{0}\text{AT}2,\text{Na}}^{\text{ISF}(\text{Neu})}$	1050	μM	Takanaga et al., 2005
ΔΨ	−70	mV	Smith et al., 1981
β	0.6^∗^	mV	Takanaga et al., 2005; Panitchob, 2015
[Na]^ISF^	141	mM	Mori et al., 2002
[Na]^Neu^	40	mM	Fedoroff and Vernadakis, 1986
V_MBEC_	3.5	μl	Mori et al., 2002; Licinio and Wong, 2009
V_ISF_	352.6	μl	Tilgmann et al., 1992; Syková et al., 2005
V_Ast_	742	μl	Ren et al., 1992; Anderova et al., 2011
V_Neu_	441.7	μl	Ren et al., 1992; Setou et al., 2004; Hosseini-Sharifabad and Nyengaard, 2007

*^∗^The details of calculation processes are reported in Taslimifar et al. (2018).*

Model calculations were performed as follows: We first determined steady state (ss) concentrations of Phe and CL in the individual NVU compartments *i* (${\left[\text{Phe}\right]}_{\text{ss}}^{\text{i}}$ and ${\left[\text{CL}\right]}_{\text{ss}}^{\text{i}}$) by prescribing constant plasma concentrations (${\left[\text{Phe}\right]}_{\text{ss}}^{\text{P}}$ and ${\left[\text{CL}\right]}_{\text{ss}}^{\text{P}}$) as model inputs and initializing the concentrations in the NVU compartments based on steady state concentration values reported in Taslimifar et al. (2018). We considered plasma Phe concentrations from 77 μM (representing normal physiologic condition) (Currie et al., 1995) to values above 1200 μM (representing sever classic PKU) (Güttler et al., 1969; Regier and Greene, 2017). Plasma CL concentration was kept constant at 739 μM (Currie et al., 1995; Bongiovanni et al., 2003).

We then prescribed fluctuating plasma Phe concentrations as

(1) $${\left[\text{Phe}\right]}_{\text{f}}^{\text{P}}={\left[\text{Phe}\right]}_{\text{ss}}^{\text{P}}\left(1+{\text{c}}_{\text{f}}\frac{{\left[\text{Phe}\right]}_{\text{δ}}^{\text{P}}}{\max\left|{\left[\text{Phe}\right]}_{\text{δ}}^{\text{P}}\right|}\right),$$

where c_f_ is a coefficient that can take on values between 0 and 1, and thereby scales the fluctuation amplitude (relative to mean), and ${\left[\text{Phe}\right]}_{\text{δ}}^{\text{P}}$ is a periodic zero-mean fluctuating component with zero initial value described by the trigonometric Fourier series

(2) $${\left[\text{Phe}\right]}_{\text{δ}}^{\text{P}}=\sum_{\text{n}=1}^{\text{N}}{\text{a}}_{\text{n}}\text{sin}\left(2{\text{πnf}}_{0}\text{t}\right)+b_{\text{n}}\text{cos}\left(2{\text{πnf}}_{0}\text{t}\right),$$

where n is an integer, f_o_ is the fundamental frequency of Phe fluctuation, and a_n_ and b_n_ are the Fourier coefficients.

For presentation purposes, we initially considered three distinct plasma Phe fluctuation profiles referred to as cases c1–c3, with values of steady state concentration, frequency and relative amplitude as given in Table 2. For each of these cases, we determined the steady state concentrations of Phe and CL (${\left[\text{Phe}\right]}_{\text{ss}}^{\text{i}}$ and ${\left[\text{CL}\right]}_{\text{ss}}^{\text{i}}$) as well as the fluctuating response (${\left[\text{Phe}\right]}_{\text{f}}^{\text{i}}$ and ${\left[\text{CL}\right]}_{\text{f}}^{\text{i}}$) in the NVU compartments. Results for this *in silico* investigation are shown as time series (excluding the initial transition from the steady state) normalized with respect to the corresponding values for normal physiologic conditions reported in Table 3 (determined for model input ${\left[Phe\right]}_{\text{ss}}^{\text{P}}$ = 77 μM, ${\left[\text{CL}\right]}_{\text{ss}}^{\text{P}}$ = 739 μM and c_f_ = 0; Currie et al., 1995; Bongiovanni et al., 2003).

**Table 2:** Fluctuation indices of exemplary Phe plasma concentration profiles.

Case number	${\left[\text{Phe}\right]}_{\text{ss}}^{\text{P}}$ (μM)	f_0_ (cycles/day)	c_f_ (−)
c1	300	1	0.99
c2	800	3	0.3
c3	1600	0.14	0.6

**Table 3:** Steady state normal physiologic (baseline) concentration values of Phe and CL in the NVU.

Compartment	Parameter	Concentration	Unit
Microvascular brain endothelial cell	${\left[\text{Phe}\right]}_{\text{ss}}^{\text{MBEC}}$	24.6 ± 146.8	μM
	${\left[\text{CL}\right]}_{\text{ss}}^{\text{MBEC}}$	235.7 ± 1408.8	μM
Brain interstitial fluid	${\left[\text{Phe}\right]}_{\text{ss}}^{\text{ISF}}$	0.03 ± 0.4	μM
	${\left[\text{CL}\right]}_{\text{ss}}^{\text{ISF}}$	0.3 ± 3.8	μM
Astrocyte	${\left[\text{Phe}\right]}_{\text{ss}}^{\text{Ast}}$	4.8 ± 40.6	μM
	${\left[\text{CL}\right]}_{\text{ss}}^{\text{Ast}}$	46.0 ± 389.7	μM
Neuron	${\left[\text{Phe}\right]}_{\text{ss}}^{\text{Neu}}$	7.0 ± 53.4	μM
	${\left[\text{CL}\right]}_{\text{ss}}^{\text{Neu}}$	46.9 ± 368.7	μM

*The details for the calculation of the steady state conditions are reported in Taslimifar et al. (2018). The plasma concentrations of Phe and CL are 77 and 739 μM, respectively (Currie et al., 1995; Bongiovanni et al., 2003).*

To further investigate the effect of fluctuations, we considered a PKU case for which we fixed the non-fluctuating plasma Phe and CL concentrations and varied the fluctuation indices (fundamental frequency and amplitude-to-mean ratio) of a purely sinusoidal signal oscillation, ${\left[\text{Phe}\right]}_{\text{δ}}^{\text{P}}$ = sin (2πf_0_t), within realistic bounds (0.14 ≤ f_0_ ≤ 7 cycles per day and 0 ≤ c_f_ ≤ 1) (Güttler et al., 1969; MacDonald et al., 1998; Ferguson and Morris, 1999; Michals-Matalon et al., 2007; Mitchell et al., 2011). We, similarly, calculated the fluctuating and steady state concentrations of Phe and CL in the NVU compartments. For each compartment, we calculated the root mean square of the difference between the fluctuating and steady state responses (normalized with respect to normal physiologic conditions) and considered it as a metric for the excursion of the concentration of Phe and CL.

Finally, we employed the model to investigate the impact of therapeutic supplementation of non-Phe LNAAs on the concentrations of Phe and CL in NVU cells. To this end, we considered the supplemented LNAAs (SL) as CL with the same kinetics, and prescribed it as constant input (representing the effective plasma concentration of SL) to the model. We then determined steady state and fluctuating responses in the NVU, considering, as model input, different values for the effective steady state concentrations of SL in the plasma (0.5, 2, and 5 mM) and of both steady state (${\left[\text{Phe}\right]}_{\text{ss}}^{\text{P}}$ = 1000 and c_f_ = 0) and sinusoidally fluctuating plasma Phe concentration (${\left[\text{Phe}\right]}_{\text{δ}}^{\text{P}}$ = sin (2πf_0_t), ${\left[\text{Phe}\right]}_{\text{ss}}^{\text{P}}$ = 1000 μM, c_f_ = 0.5 and f_0_ = 1 cycles/day). We show the responses as time series (excluding transition from steady state) normalized with respect to the corresponding values of baseline physiologic conditions.

### Sensitivity Analysis

We evaluated the sensitivity of the reported results on the choice of literature-reported model parameter values. To this end, we calculated model output for 100 cases in which the nominal model input parameters [maximum transport rates, Michaelis–Menten binding constants and steady state physiologic concentration values of LNAAs in individual compartments (Tables 1, 3)] were varied randomly in a range of ±20% from their respective nominal values. In the results, the shown upper and lower bounding curves correspond to standard deviations of the calculated concentrations from those computed under nominal parameter conditions.

## Results

### NVU Steady State Condition in Relation to Steady State Plasma Phe Concentration

**FIGURE 2:**
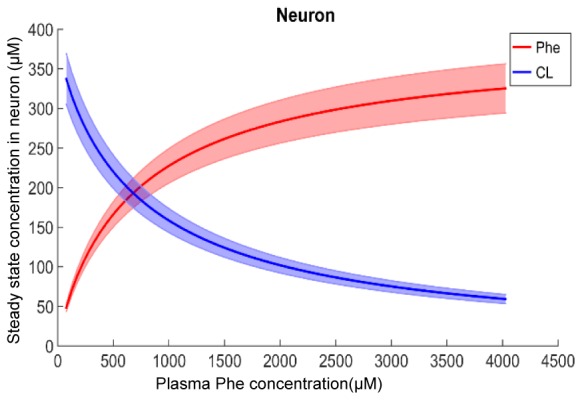
Model calculation of the steady state concentration of Phe and CL in neurons in relation to Phe concentration in the plasma. The bold solid lines correspond to results obtained with nominal model parameter values. The lower and upper bounds indicate standard deviation determined by sensitivity analysis (see section Materials and Methods). Similar relations between Phe and CL steady state concentrations are also observed in the other NVU compartments (see Supplementary Figure S1).

Figure 2 shows plots of steady state concentrations of Phe and CL in neurons versus steady state concentrations of plasma Phe from 77 μM (normal physiologic conditions) to values above 1200 μM (severe classic PKU). The reason why AA levels in the modeled neuron reach equilibrium (even though the Na^+^ electrochemical force drives influx through B^0^AT2) is their low extracellular concentration in the brain interstitial fluid. Phe and CL concentrations in the other compartments are shown in Supplementary Figure S1. In all NVU compartments, Phe concentration increases with its plasma concentration, while the opposite behavior is observed for CL. In Supplementary Figure S1E, we show the steady state responses in the whole brain tissue as volume-weighted average of the steady state responses in the individual NVU compartments. Given that animal species are different in terms of NVU geometry and also kinetic characteristics of LNAA transporters (Smith et al., 1987; Hargreaves and Pardridge, 1988; Martynyuk et al., 2010), a 1:1 comparison between species cannot be provided for concentration behavior of LNAA in the brain. However, the observed growth trend we determined for Phe in whole brain tissue in our *in silico* adult PKU rat brain model is qualitatively in line with experimental observations in a PKU mouse model (van Vliet et al., 2015; Belanger et al., 2018).

### The Dynamics of LNAAs in the NVU Are Influenced by Phe Concentration Fluctuations in the Plasma

**FIGURE 3:**
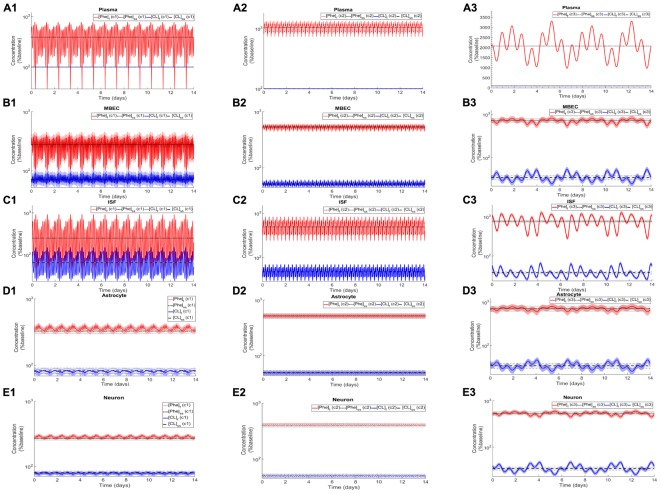
Dynamic changes in the concentrations of Phe and CL in MBECs, ISF, astrocytes and neurons in response to fluctuations of Phe concentration in the plasma. Panel **(A)** shows the plasma concentration of L-phenylalanine (Phe) and competing LNAAs (CL) for the three cases c1, c2, and c3 described in the Methods. For each case, panels **(B1–B3,C1–C3,D1–D3,E1–E3)** show, respectively, the computed steady state concentrations of Phe and CL concentrations ([Phe]_ss_ and [CL]_ss_) as well as the fluctuating response ([Phe]_f_ and [CL]_f_) in MBECs, ISF, astrocytes and neurons. The lower and upper bounds indicate standard deviation determined by sensitivity analysis (see section Materials and Methods). The physiologic baseline concentrations of Phe and CL are reported in Table 3.

After the assessment of the NVU steady state response to steady state plasma Phe concentrations, we calculated the dynamic changes of Phe and CL in the NVU in response to plasma Phe fluctuations. Figure 3 shows the evolution of the concentrations of Phe and CL over time in MBECs (Figure 3B1–B3), brain ISF (Figure 3C1–C3), astrocytes (Figure 3D1–D3), and neurons (Figure 3E1–E3) for the three plasma Phe fluctuation cases c1, c2, and c3 (Figure 3A1–A3) (normalized with respect to normal physiologic conditions) over a span of 14 days. The shown upper and lower bounding curves correspond to standard deviations obtained through sensitivity analysis (see section Materials and Methods). In all cases, the results highlight the competition between Phe and CL: for example, the transfer of Phe through MBEC via the antiporter LAT1 is associated with movements of CL in reverse direction at both luminal and abluminal BBB membranes (Figure 3B1–B3). Once Phe and CL gain entry into the brain ISF (Figure 3C1–C3), they are either exchanged back into MBECs through abluminal LAT1 (Figure 3B1–B3) and/or astrocytes via LAT2 (Figure 3D1–D3), and/or co-transported along with sodium ions into neurons via B^0^AT2 (Figure 3E1–E3). Additionally, in all cases, the steady state Phe concentration in brain ISF, astrocyte and neurons are characterized by values higher than under normal physiologic conditions (which correspond to “baseline” or 100% in the figure), while the opposite is observed for CL concentrations. We also observe that the concentration dynamics of both Phe and CL in the NVU compartments are influenced by plasma Phe fluctuations, with the impact dependent on plasma Phe fluctuation frequency, mean value, and amplitude-to-mean ratio. For example, in case c1, plasma Phe fluctuations strongly affect the dynamics of Phe and CL concentration in brain ISF and MBECs and, to a lesser extent, in astrocytes and neurons. In case c2, the variations in concentrations of Phe and CL in astrocyte and neurons are not significant as compared to corresponding changes in MBECs and brain ISF. Finally, in case c3, we observe large variations in concentrations of Phe and CL in brain ISF and MBEC, but comparably lower variations in astrocytes and neurons.

### Association Between Plasma Phe Fluctuation and Changes in Phe and CL Concentration in the NVU

To study how perturbations in Phe and CL concentrations in the NVU relate to plasma Phe fluctuation indices, we simultaneously varied f_0_ and c_f_ of sinusoidally fluctuating plasma Phe concentration, and then calculated the corresponding changes in concentration of Phe and CL in the NVU compartments. We thereby quantified the associations between Phe and CL excursion and the plasma Phe frequency and amplitude-to-mean ratio. Supplementary Figure S2 shows corresponding results observed over a span of 14 days (${\left[\text{Phe}\right]}_{\text{δ}}^{\text{P}}$ = sin (2πf_0_t) and ${\left[\text{Phe}\right]}_{\text{ss}}^{\text{P}}$ = 1600 μM). The largest Phe and CL changes are observed in all NVU compartments for large amplitude-to-mean ratio of Phe fluctuations in the plasma. Additionally, low frequencies lead to higher excursion for Phe and CL in astrocytes and neurons and also in ISF for CL, whereas in the same ISF compartment the Phe excursions are lower under low frequencies (Supplementary Figures S2B1,2,C1,2,D1,2). Finally, we observed that in MBECs, excursion levels are not influenced by the fluctuation frequency and that the changes in Phe and CL concentration are also not significantly sensitive to fluctuation frequency in the other compartments when the fluctuation coefficient is small (Supplementary Figures S2A1–D2).

### Impact of LNAA Supplementation on Phe and CL Concentrations in the NVU

**FIGURE 4:**
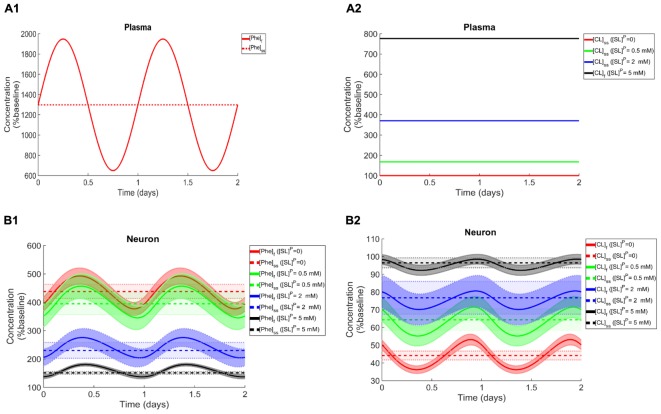
Impact of LNAA supplementation on Phe and CL concentrations in the NVU. Panels **(A1,A2)** show the plasma concentration of L-phenylalanine (Phe) and competing LNAAs (CL) used as model input. Panels **(B1,B2)** show the model calculations for steady state concentrations of Phe and CL ([Phe]_ss_ and [CL]_ss_) as well as the fluctuating response ([Phe]_f_ and [CL]_f_) in the neuronal compartment for various concentration levels of supplemented LNAAs in the plasma ([SL]^P^). The lower and upper bounds indicate standard deviation determined by sensitivity analysis (see section Materials and Methods). In all panels, the baseline physiologic concentrations of Phe and CL are reported in Table 3.

To elucidate the impact of non-Phe LNAA supplementation, we determined Phe and CL concentrations in neurons (Figure 4B1,B2), MBECs (Supplementary Figures S3B1,B2), ISF (Supplementary Figures S3C1,C2) and astrocytes (Supplementary Figures S3D1,D2) in response to various concentrations of SL in the plasma under both steady state and fluctuating Phe conditions (Figure 4A1,A2). The shown upper and lower bounding curves correspond to standard deviations investigated by performing sensitivity analysis. Our results show that supplementation of LNAA attenuates the disturbed steady state concentration of Phe and CL in the NVU and modulates their concentrations toward physiologic baseline conditions (Table 3). This is because SL together with CL increases the competitions between Phe and non-Phe LNAAs through LAT1 across the BBB and thereby inhibits LAT1 mediated trans-endothelial transport of Phe fluctuations from plasma into the brain. As a consequence, this leads to a reduction in the amplitude level of Phe and CL fluctuations in neurons, MBECs, ISF and astrocytes (Figure 4B1,B2 and Supplementary Figures S3B1,B2,C1,C2,D1,D2, respectively). In addition, the increased competition via LAT1 leads to delayed response in neurons, ISF and astrocytes (Figure 4B1,B2 and Supplementary Figures S3C1,C2,D1,D2, respectively).

## Discussion

Failure of AA homeostasis in general negatively affects cell function (Suraweera et al., 2012). In cells of the NVU, Phe and CL are involved in a variety of processes, including the synthesis of essential neurotransmitters such as dopamine (3-hydroxytyramine) and serotonin (5-hydroxytryptamine; 5-HT) (Fernstrom, 1994, 2005; Lieberman, 2000; Cansev and Wurtman, 2007), and the production of proteins and lipids (e.g., myelin) (Sperringer et al., 2017; Yudkoff, 2017), although their function in the latter processes is not fully understood. Hence, depending on the full set of roles of Phe and CL, oscillations of their concentrations in the NVU could have a negative impact on central nervous system function. However, fluctuating NVU-LNAA concentrations are difficult to measure in animal models of PKU. Furthermore, in patient studies, the effects of elevated steady state plasma Phe concentration cannot be easily differentiated from those caused by increased fluctuations in Phe. The here introduced *in silico* model can serve as an alternative tool to probe these complex dynamics quickly and quantitatively.

We first showed that with increasing Phe concentration in plasma, the steady state concentration of Phe in the NVU and in whole brain increases, while CL concentration decreases. The growth trend we determined for Phe in our *in silico* adult PKU rat brain model is qualitatively in line with experimental observations in a PKU mouse model (van Vliet et al., 2015). We then showed that the propagation of plasma Phe concentration oscillations into the NVU largely depend on fluctuation indices such as frequency and amplitude-to-mean ratio. We observed the largest Phe and CL concentration excursions in the NVU for low frequencies and large amplitude-to-mean ratios of plasma Phe fluctuations. Additionally, we observed that, for the small fluctuation coefficients, the sensitivity of Phe and CL excursions to plasma Phe fluctuation frequency is not significant. Finally, we showed that supplementation of LNAAs not only reduces steady state concentrations in the NVU toward normal physiologic values, but also leads to a reduction in Phe and CL fluctuation amplitude. In conclusion, our *in silico* experiments support LNAA supplementation as therapeutic strategy for reducing the propagation of plasma Phe concentration fluctuations into the brain. The model itself can also be seen as a potential stepping-stone for the development of a patient-specific LNAA supplementation treatment-planning tool.

The computational model has been built with a number of simplifying assumptions. In particular, we focused on the pathways mediated by dominant transporters and thus disregarded pathways related to metabolism, diffusion and NVU transporters with low levels of expression, which have been shown to be of lesser importance (Sadasivudu and Lajtha, 1970; Smith and Takasato, 1986). Additionally, we considered Phe and CL as a homogenous mixture within the individual NVU compartments. Furthermore, we considered CL as a single entity rather than accounting one by one for each individual competitor, i.e., for L-isoleucine, L-tyrosine and others. The validity of these assumptions are discussed in more detail in Taslimifar et al. (2018). To account for effects of uncertainties associated with the values of the model parameters, we have performed a sensitivity analysis demonstrating that our conclusions are robust with respect to reasonable parameter variations.

## Summary

Using a computational model of adult rat NVU-LNAA homeostasis, we investigated the effects of plasma Phe fluctuations on the dynamics of LNAAs in MBECs, brain ISF, astrocytes and neurons in PKU. Comparable *in vivo* investigations are not readily doable with current techniques. Our results show that plasma Phe fluctuations can propagate into the NVU and change there the concentration of LNAAs, with the magnitude of this effect largely dependent on the frequency and amplitude-to-mean ratio of the plasma concentration fluctuations. Finally, we quantified the therapeutic impact of supplementation of LNAAs in attenuating the disturbed fluctuating and steady state concentrations of Phe and CL in the cells of NVU in PKU disorder toward normal physiologic levels.

## Author Contributions

MT implemented the computational model and performed the calculations with SB. FV and VK directed the research. All authors conceived and designed the study, analyzed the data, wrote the manuscript, and approved the final version.

## Conflict of Interest Statement

The authors declare that the research was conducted in the absence of any commercial or financial relationships that could be construed as a potential conflict of interest.
