# Supplementary material for: Propagation of Plasma L-Phenylalanine Concentration Fluctuations to the Neurovascular Unit in Phenylketonuria: An in silico Study
Source: Front Physiol. 2019 Apr 2;10:360. doi: 10.3389/fphys.2019.00360 (PMC6454150; doi:10.3389/fphys.2019.00360)
Supplement: Supplementary file 1 [file Data_Sheet_1.PDF]

*Supplementary Material*

# Propagation of plasma L-phenylalanine concentration fluctuations to the neurovascular unit in phenylketonuria: An in silico study.

**Mehdi Taslimifar[1,2], Stefano Buoso[1,3], Francois Verrey[2,4,5†], Vartan Kurtcuoglu[1,4,5,6*†]**

[1] The Interface Group, Institute of Physiology, University of Zurich, Switzerland;

[2] Epithelial Transport Group, Institute of Physiology, University of Zurich, Switzerland;

[3] Institute for Diagnostic and Interventional Radiology, Zurich University Hospital, Zurich, Switzerland;

[4] Zurich Center for Integrative Human Physiology, University of Zurich, Switzerland;

[5] National Center of Competence in Research, Kidney.CH, Switzerland;

[6] Neuroscience Center Zurich, University of Zurich, Zurich, Switzerland.

[†] contributed equally.

*** Correspondence:** Vartan Kurtcuoglu

Institute of Physiology

University of Zürich

Winterthurerstrasse 190,

CH-8057 Zürich, Switzerland

Telephone: +41 (0) 44 635 50 55

Email: vartan.kurtcuoglu@uzh.ch

# Figure S1

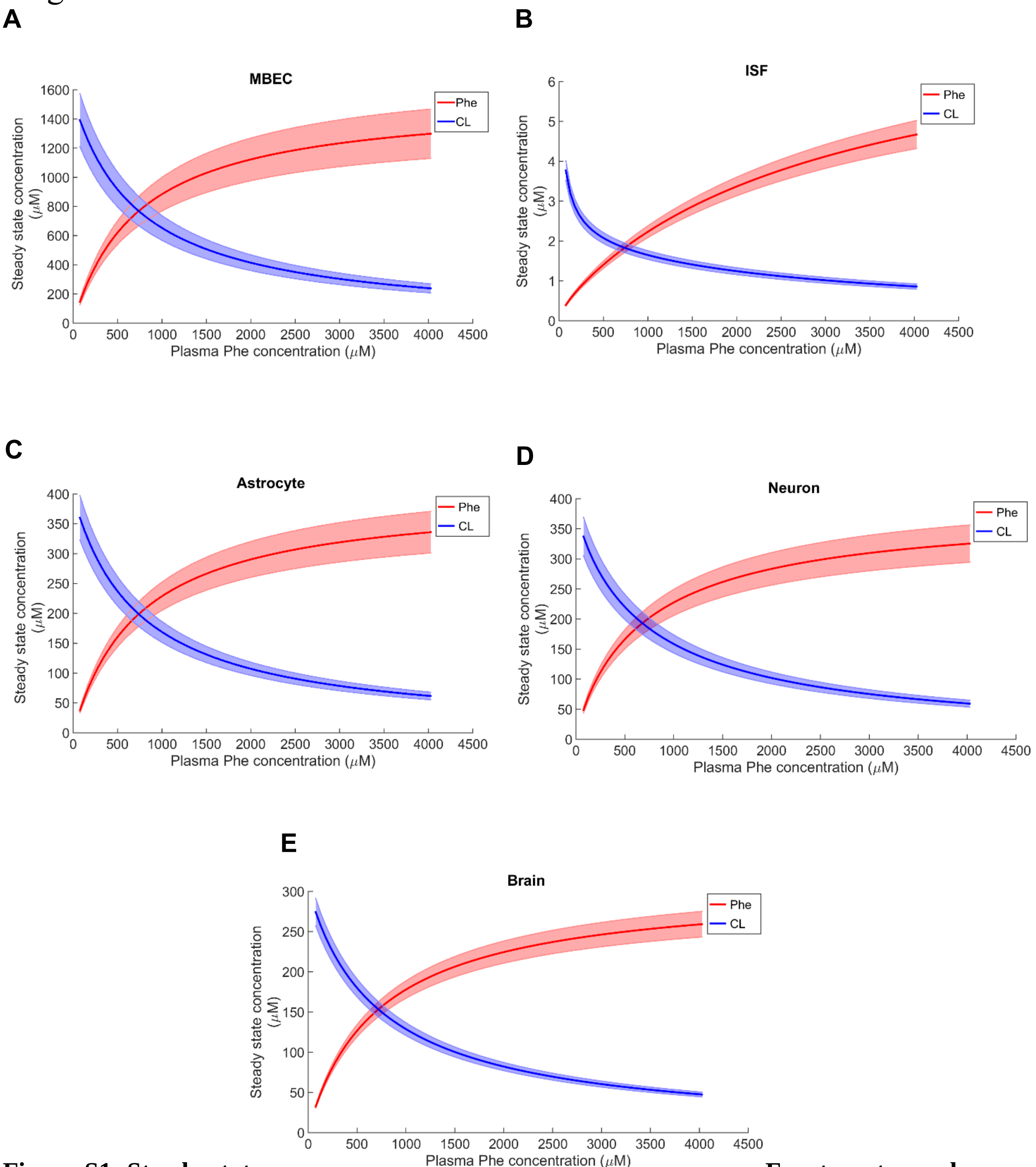


**Figure S1. Steady state concentration of Phe and CL in MBECs, ISF, astrocytes and neurons and whole brain in relation to Phe concentration in the plasma.** Panels A to E show model calculations for the steady state concentrations of Phe and CL in MBECs (panel A), ISF (panel B), astrocytes (panel C), neurons (panel D) and the whole brain (panel E). The bold solid lines correspond to results obtained with nominal model parameter values. The lower and upper bounds indicate standard deviation determined by sensitivity analysis (see Methods).

# Figure S2

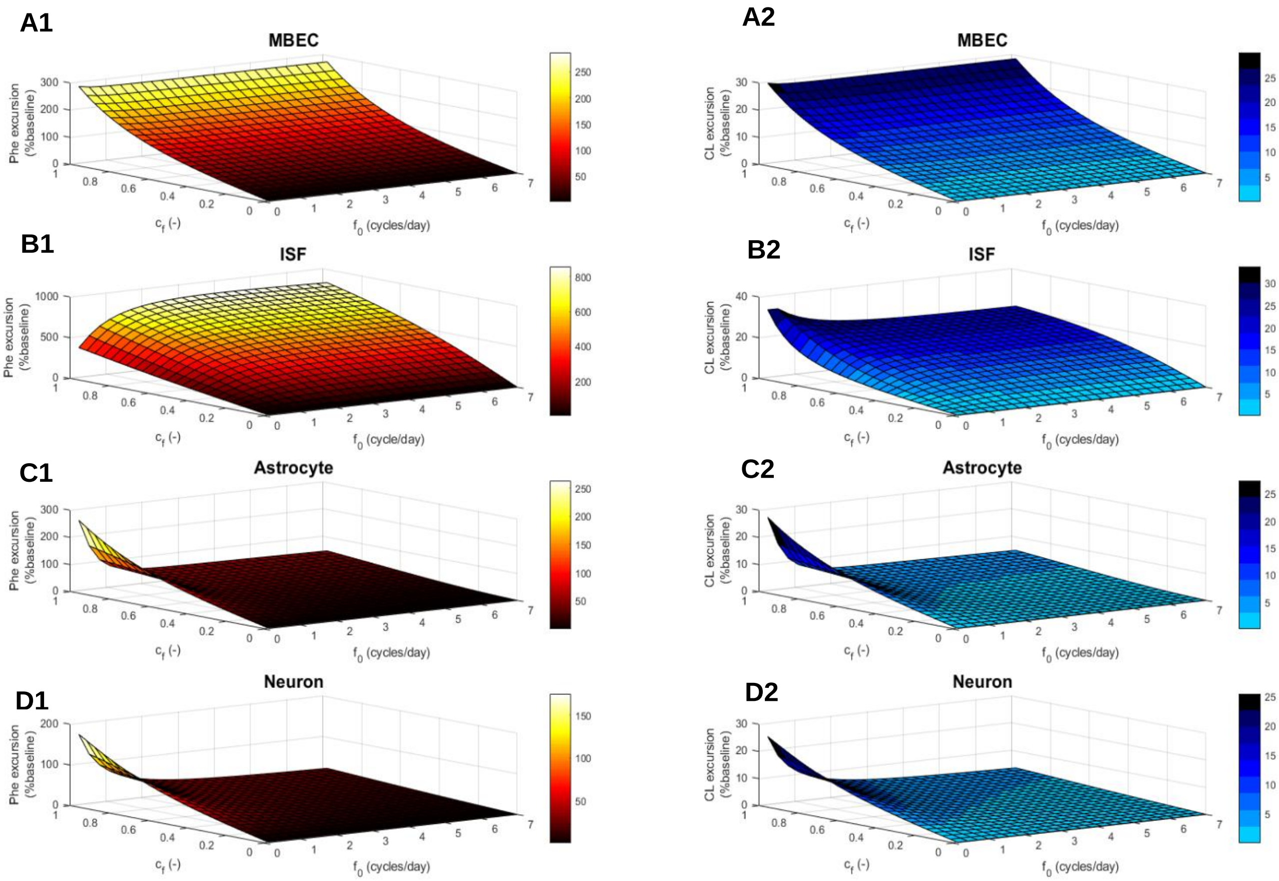


**Figure S2. Phe and CL excursion in the NVU compartments in relation to the plasma Phe fluctuation indices.** Panels A to D show model calculations for Phe (left column) and CL excursion (right column) in the NVU compartments as a function of fundamental frequency ($f_0$) and amplitude-to-mean ratio ($c_f$) of Phe in the plasma (see Methods for definition of 'excursion'). The results are normalized with respect to normal physiologic concentrations (baseline).

# Figure S3

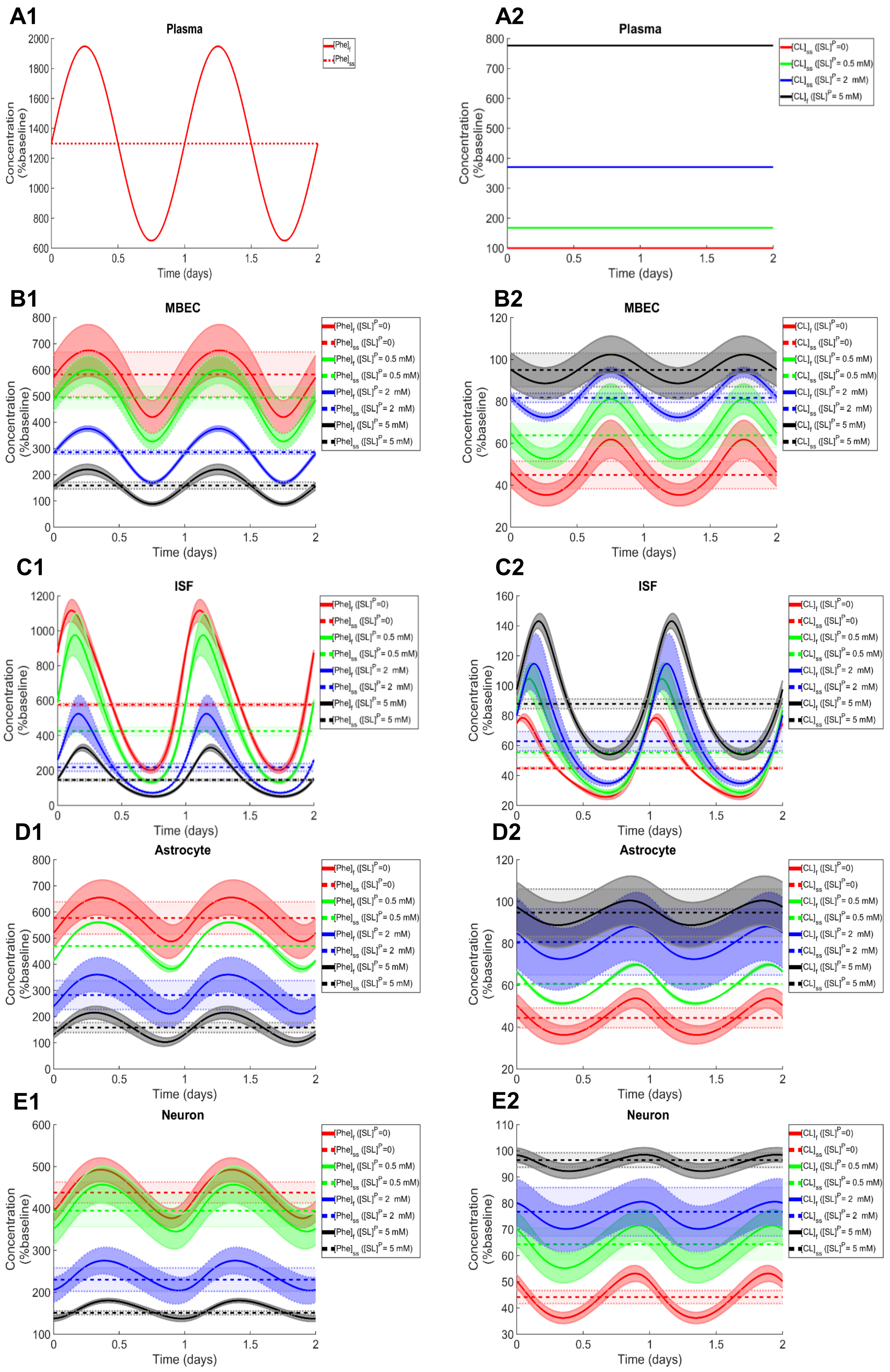
A1
Plasma
Concentration (%baseline)
Time (days)
[Phe]f
[Phe]ss
A2
Plasma
[CL]ss ([SL]P=0)
[CL]ss ([SL]P= 0.5 mM)
[CL]ss ([SL]P= 2 mM)
[CL]f ([SL]P= 5 mM)
B1
MBEC
B2
MBEC
C1
ISF
C2
ISF
D1
Astrocyte
D2
Astrocyte
E1
Neuron
E2
Neuron
[Phe]f ([SL]P=0)
[Phe]ss ([SL]P=0)
[Phe]f ([SL]P= 0.5 mM)
[Phe]ss ([SL]P= 0.5 mM)
[Phe]f ([SL]P= 2 mM)
[Phe]ss ([SL]P= 2 mM)
[Phe]f ([SL]P= 5 mM)
[Phe]ss ([SL]P= 5 mM)
[CL]f ([SL]P=0)
[CL]ss ([SL]P=0)
[CL]f ([SL]P= 0.5 mM)
[CL]ss ([SL]P= 0.5 mM)
[CL]f ([SL]P= 2 mM)
[CL]ss ([SL]P= 2 mM)
[CL]f ([SL]P= 5 mM)
[CL]ss ([SL]P= 5 mM)

**Figure S3. Impact of LNAA supplementation on Phe and CL concentrations in the NVU.** Panels (A1, 2) show the plasma concentration of L-phenylalanine (Phe) and competing LNAAs (CL) used as model input. Panels (B1, 2), (C1, 2), (D1, 2) and (E1, 2) show the model calculations for steady state concentrations of Phe and CL ($[\mathrm{Phe}]_{\mathrm{ss}}$ and $[\mathrm{CL}]_{\mathrm{ss}}$) as well as the fluctuating response ($[\mathrm{Phe}]_{\mathrm{f}}$ and $[\mathrm{CL}]_{\mathrm{f}}$) in the NVU compartments for various concentration levels of supplemented LNAAs in the plasma ($[\mathrm{SL}]^{\mathrm{P}}$). The lower and upper bounds indicate standard deviation determined by sensitivity analysis (see Methods). In all panels, the baseline physiologic concentrations of Phe and CL are reported in Table 3.
